# An Intelligent Mobile-Enabled System for Diagnosing Parkinson Disease: Development and Validation of a Speech Impairment Detection System

**DOI:** 10.2196/18689

**Published:** 2020-09-16

**Authors:** Liang Zhang, Yue Qu, Bo Jin, Lu Jing, Zhan Gao, Zhanhua Liang

**Affiliations:** 1 International Business College Dongbei University of Finance and Economics Dalian China; 2 School of Innovation and Entrepreneurship Dalian University of Technology Dalian China; 3 Department of Neurology The First Affiliated Hospital of Dalian Medical University Dalian China; 4 Beijing Haoyisheng Cloud Hospital Management Technology Ltd Beijing China

**Keywords:** Parkinson disease, speech disorder, remote diagnosis, artificial intelligence, mobile phone app, mobile health

## Abstract

**Background:**

Parkinson disease (PD) is one of the most common neurological diseases. At present, because the exact cause is still unclear, accurate diagnosis and progression monitoring remain challenging. In recent years, exploring the relationship between PD and speech impairment has attracted widespread attention in the academic world. Most of the studies successfully validated the effectiveness of some vocal features. Moreover, the noninvasive nature of speech signal–based testing has pioneered a new way for telediagnosis and telemonitoring. In particular, there is an increasing demand for artificial intelligence–powered tools in the digital health era.

**Objective:**

This study aimed to build a real-time speech signal analysis tool for PD diagnosis and severity assessment. Further, the underlying system should be flexible enough to integrate any machine learning or deep learning algorithm.

**Methods:**

At its core, the system we built consists of two parts: (1) speech signal processing: both traditional and novel speech signal processing technologies have been employed for feature engineering, which can automatically extract a few linear and nonlinear dysphonia features, and (2) application of machine learning algorithms: some classical regression and classification algorithms from the machine learning field have been tested; we then chose the most efficient algorithms and relevant features.

**Results:**

Experimental results showed that our system had an outstanding ability to both diagnose and assess severity of PD. By using both linear and nonlinear dysphonia features, the accuracy reached 88.74% and recall reached 97.03% in the diagnosis task. Meanwhile, mean absolute error was 3.7699 in the assessment task. The system has already been deployed within a mobile app called No Pa.

**Conclusions:**

This study performed diagnosis and severity assessment of PD from the perspective of speech order detection. The efficiency and effectiveness of the algorithms indirectly validated the practicality of the system. In particular, the system reflects the necessity of a publicly accessible PD diagnosis and assessment system that can perform telediagnosis and telemonitoring of PD. This system can also optimize doctors’ decision-making processes regarding treatments.

## Introduction

Parkinson disease (PD) is a long-term degenerative disorder of the central nervous system that mainly affects the motor system. In the early stages, the symptoms include tremor; rigidity; slowness of movement; and difficulty with walking, talking, thinking, or completing other simple tasks. Dementia becomes common in the later stages of the disease. More than a third of patients have experienced depression and anxiety [1]. Other symptoms include sensory and sleep problems. In 2017, PD affected more than 10 million people worldwide, making it the second-most common neurological condition after Alzheimer disease. Currently, there is no cure for PD [2]. Accurate diagnosis, prognosis, and progression monitoring remain nontrivial.

As reported in previous work [3,4], approximately 90% of patients with PD develop voice and speech disorders during the course of the disease, which can have a negative impact on functional communication, thus leading to a decline in the quality of life [5]. Reduced volume (ie, hypophonia), reduced pitch range (ie, monotone), and difficulty with the articulation of sounds or syllables (ie, dysarthria) are the most common speech problems [6]. At the same time, many patients gradually dislike communication because of their own language barriers, which will cause more serious speech disorders and then form a vicious circle. Note that the speech signal–based test is noninvasive and can be self-administered. Hence, it has been regarded as a promising approach in PD diagnosis, evaluation, and progression monitoring, especially in the telediagnosis and telemonitoring medical fields.

In this work, we built a publicly accessible real-time system to efficiently diagnose and assess the severity of PD via speech signal analysis. The most relevant works can be found in Lahmiri et al [7] and Wroge et al [8]. They utilize similar machine learning algorithms as those based on previously proposed audio features [9-13]; however, their work neither considered severity assessment of PD nor made a publicly accessible app that allows for real-time mobile-aided PD diagnosis or evaluation, which is actually a trend and even a necessity in the current 4G and future 5G era for telediagnosis and telemonitoring. For instance, the outbreak of coronavirus disease 2019 (COVID-19) highlights the importance of intelligent and accurate telehealth during disease epidemics.

More specifically, our system first collects the speech signals of the subjects and then utilizes speech signal processing techniques to extract a variety of speech impairment features; it further utilizes advanced machine learning algorithms to diagnose PD and analyze the disease severity. In our work, in the speech signal feature-extraction stage, we utilized many traditional and novel methods to obtain clinically meaningful voice signal features, such as jitter, fine-tuning, recurrence period density entropy, pitch period entropy, signal-to-noise ratio, harmonics-to-noise ratio (HNR), and the mel frequency cepstral coefficient [9-11]. We regarded the PD diagnosis task as a classification problem and then utilized classical algorithms (eg, support vector machine [SVM] and artificial neural network [ANN]) to perform diagnosis. We formed the PD severity assessment task into a regression problem, with the Unified Parkinson Disease Rating Scale (UPDRS) score as the dependent variable; the UPDRS is the most widely employed scale for tracking PD symptom progression. Various regression algorithms (eg, support vector regression [SVR] and least absolute shrinkage and selection operator [LASSO] regression) were tested. We then obtained the most suitable model by comparing and blending different algorithms. In the end, we developed a mobile phone app for our system to realize remote diagnosis, severity evaluation, and progression monitoring of PD, which will significantly reduce detection and prevention costs.

The main structure of this paper is divided into four parts: (1) description of the methods used in our system: data collection, data preprocessing, feature extraction of speech signals, classification, and regression problem formulation, (2) analysis of our experimental results, (3) system description of our mobile app, and (4) final discussion.

## Methods

### Data Collection

The speech signal data used in the experiment came from two sources:

1. One part of the dataset came from the open data platform from the University of California Irvine (UCI) Machine Learning Repository, where three sets of parkinsonian speech data with different characteristics were obtained.
2. The other part of the dataset was collected in collaboration with the Department of Neurology, the First Affiliated Hospital of Dalian Medical University, China. The data recorded the voice signals of patients with PD.

In practice, the collected pronunciation content needs to be short and reflect the patient's speech disorder to a certain extent. On one hand, considering the need for different languages, dialects, and accents as well as unclear pronunciations, we adopted the continuous pronunciation method. Meanwhile, the control of the vocal cords and airflow is also weakened due to the weakening control of the pronunciation system of the nervous system. On the other hand, since the relationship between the vibration of the vocal cords and the speech disorder is relatively strong, the vowels can better reflect the degree of speech impairment [6,11,14]. Another fact is that the basic vowels in different regions of the world are very similar, so it is more reasonable to use vowels. The vowels used here are the five long vowels with the following English phonetic symbols: [ɑ:], [ɜ:], [i:], [ɔ:], and [u:]; the subjects are required to pronounce them repeatedly. The collected syllables are shown in Table 1.

**Table 1:** Collected syllables.

International phonetic symbol	Duration (seconds)
[ɑ:]	3
[З:]	3
[i:]	3
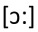	3
[u:]	3

The UPDRS [15] is the most commonly used severity indicator in clinical studies of PD. It is evaluated via filling out a form, which requires considerable medical expertise, so it is difficult for patients to perform self-testing using this scale. That explains why we need automatic and artificial intelligence–powered prediction tools. We collected the UPDRS score as the dependent variable in our regression task. At present, UPDRS version 3.0 is the most widely used version, and it can be divided into four parts:

1. Mentation, behavior, and mood, including a total of four questions (16 points).
2. Activities of daily living, including a total of 13 questions (52 points).
3. Motor examination, including a total of 14 questions (108 points).
4. Treatment complications, including a total of 11 questions (23 points).

In summary, UPDRS version 3.0 has a total of 42 questions and the highest score is 199 points. The higher the UPDRS score, the more serious the PD is. The third item, *motor examination*, can reflect the severity of speech disorder. In practice, when collecting the data, the doctor is required to evaluate the total UPDRS score as well as the value of the motor examination score.

Note that the first part of the data is open source, and we can easily download this data from UCI's official website. Therefore, the datasets were mainly used to train the machine learning models and verify the validity of the dysphonia features, all of which have been integrated in our app system. This part of the data will be introduced in detail in the Results section. The second part of the data requires us to work closely with local hospitals—we collected PD patients’ vocal data in a local hospital; the data collection table is shown in Multimedia Appendix 1. The Data Preprocessing section that follows describes how we processed the second part of the data, which has been implemented as a function in our app. We then extracted the dysphonia features, which have also been integrated as a function in our app system. Moreover, we tested them with machine learning models, which have been trained based on the first part of data, and achieved good results in the PD diagnosis task. Until now, the number of collected Chinese speech signals is still not big enough to train an effective model. Therefore, our model within our app system was trained by the first part of the data: the first and third datasets were used in the diagnosis task and severity assessment task, respectively. However, this app is continuously collecting new data, including positive and negative samples. As the amount of data increases in the future, we will utilize advanced technology, such as transfer learning, to realize PD diagnosis and severity evaluation for people in various regions.

### Data Preprocessing

The initially collected voice signals cannot be directly used; some preprocessing was required. This operation removed some of the interference factors and paved the way for subsequent feature extraction.  The formats of different audio files were unified into the WAV file format, with 44,100 Hz sampling frequency and two channels. These audio files were then uploaded into the back-end server for storage.

The first step of data preprocessing is *sampling frequency conversion*, that is, resampling, which can uniformly record the speech frequency and reduce the amount of calculation by down-clocking. In our work, only one channel of the speech signal (ie, the left channel) is reserved, and then the sampling frequency is converted to 10 kHz.

**Figure 1:**
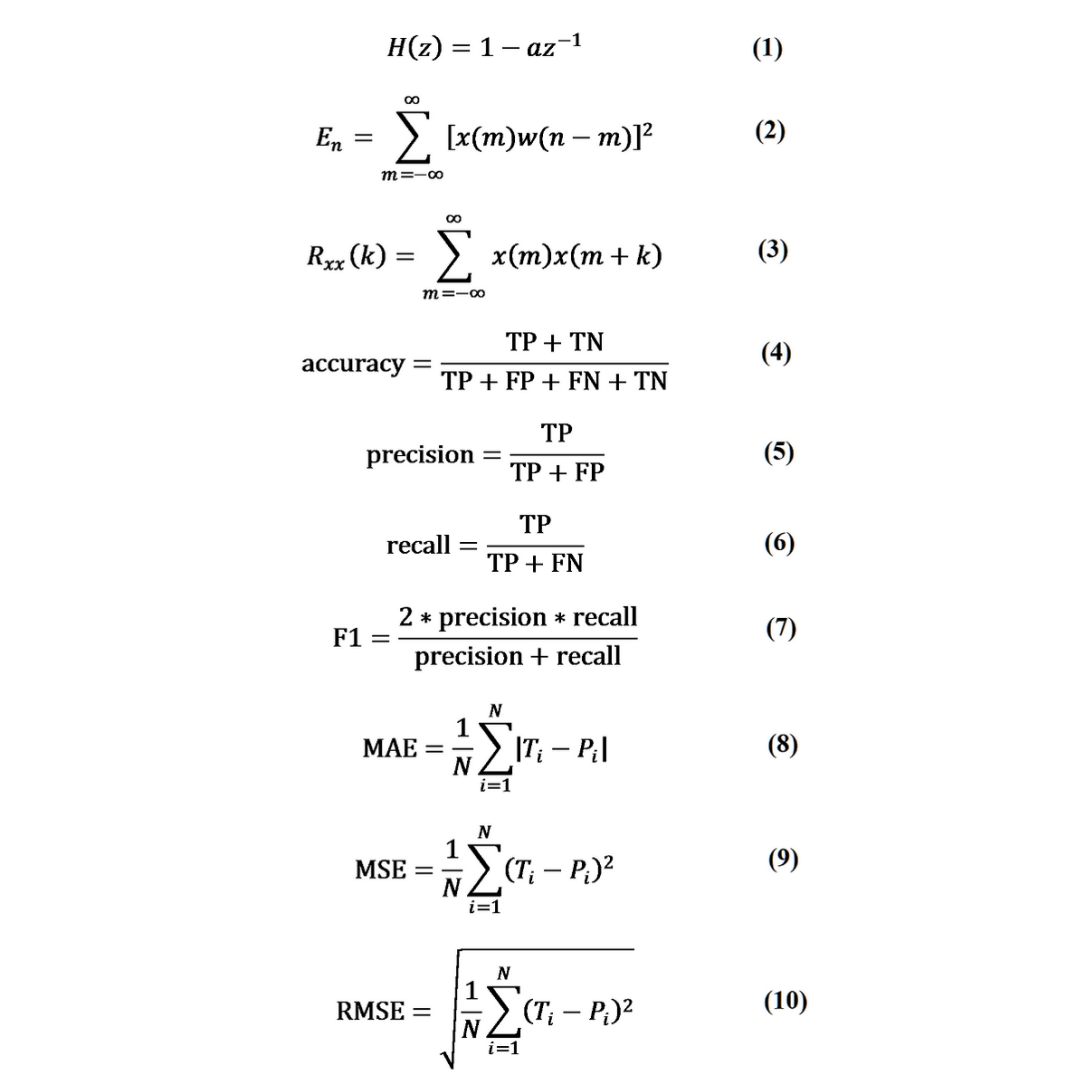
Equations 1-10. FN: false negative; FP: false positive; MAE: mean absolute error; MSE: mean square error; RMSE: root mean square error; TN: true negative; TP: true positive.

The second step is *pre-emphasis*. Since the low-frequency part of speech signals tends to contain noise, we performed pre-emphasis to filter out the low frequencies and improve the resolution of the high-frequency part of speech signals. In our work, a first-order, finite impulse response, high-pass digital filter was used to achieve pre-emphasis [16]. The transfer function is defined in equation 1 of Figure 1. In equation 1, *a* is the pre-emphasis coefficient; generally, 0.9 < *a* < 1.0. Let *x*(*n*) denote the voice sample value at time *n*. After the pre-emphasis processing, the result is *y*(*n*) = *x*(*n*) – *ax*(*n*–1), where *a*=0.9375.

The third step is *windowing and framing*. The speech signal was divided into some shorter signal segments (ie, frames) for processing, which is the framing process, such that the signal can be treated as stationary in the short-time window. In practice, to reduce the impact of segmenting on the statistical properties of the signal, we applied windowing to the temporal segments. The frame width in our work was set as 25 milliseconds long, the frame shift was 10 milliseconds long, and the Hamming window was leveraged as the window function.

The fourth step is *silent discrimination*. Because there is no guarantee that the collected audio files will always have sound, it is necessary to filter out the blank periods of those sounds. Therefore, silent discrimination, also known as voice endpoint detection, was required. A common solution is to use double-threshold methods [17], which are based on the principles of short-time energy, short-term average amplitude, and short-time zero-crossing rate. In our work, for the sake of simplicity and algorithm efficiency, we utilized only short-term energy as the principle for the double-threshold method. The definition of short-term average energy is shown Figure 1, equation 2.

**Figure 2:**
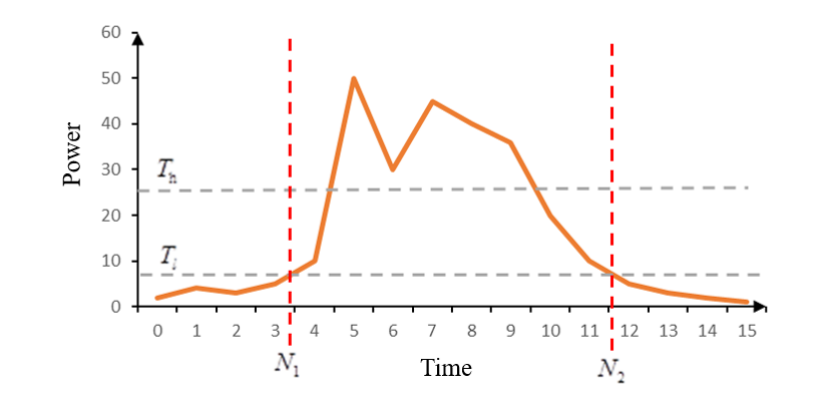
Principle of the double-threshold method. *N*_1_: starting point; *N*_2_: ending point; *T_h_*: upper threshold; *T_l_*: lower threshold.

For illustration, as is shown in Figure 2, we let *T_h_* and *T_l_* denote the upper and lower thresholds, respectively. The voiced part must have a section above *T_h_*. The endpoint energy of the voiced part is equal to *T_l_*. *N*_1_ is the starting point, *N*_2_ is the ending point, and *w* is the Hamming window. The fifth step is *fundamental frequency extraction*. The fundamental frequency refers to the lowest and theoretically strongest frequency in the sound, which reflects the vibration frequency of the sound source. In our work, we adopted the most widely used autocorrelation method to extract the fundamental frequency.

The short-term autocorrelation function is defined in Figure 1, equation 3. We need to obtain the first positive peak point, *R_xx_*(*k_f_*), after crossing the zero point in sequence *R_xx_*(*k*), and 1/*k_f_* is the extracted fundamental frequency.

Note that the audio files may be mixed with unknown noise, which can cause a sudden jump at some points. These points are called wild points or outliers. Therefore, it is necessary to initially remove the wild points. We first calculated the average value of the fundamental frequency of the audio and then deleted the point that was too far from the average value.

### Dysphonia Features

In 2012, Tsanas et al summarized 132 features of speech impairments [11]. Considering the speed requirement of the real-time system, the selected model cannot use all of the features. The final selected features [18-23] are illustrated in Table 2.

**Table 2:** Dysphonia features.

Classification and dysphonia features		Description
**Pitch [18] (fundamental frequency)**		
	*F*_0_*_*mean	Mean of pitch
	*F*_0_*_*max	Max of pitch
	*F*_0_*_*min	Min of pitch
	*F*_0_*_*median	Median of pitch
	*F*_0_*_*std	SD of pitch
**Jitter [18] (pitch period perturbation)**		
	Jitter	Jitter
	Jitter_abs	Absolute jitter
	Jitter_PPQ5	5 adjacent points’ jitter
	Jitter_rap	3 adjacent points’ jitter
	Jitter_ddp	Difference of 3 adjacent points’ jitter
**Shimmer [18] (amplitude perturbation)**		
	Shimmer	Shimmer: percentage
	Shimmer_dB	Shimmer: decibels (dB)
	Shimmer_APQ5	5 adjacent points’ shimmer
	Shimmer_APQ3	3 adjacent points’ shimmer
	Shimmer_dda	Difference of 3 adjacent points’ shimmer
	Shimmer_APQ11	11 adjacent points’ shimmer
**Harmonics-to-noise ratio (HNR) and noise-to-harmonics ratio (NHR) [19]**		
	HNR_mean	Mean of HNR
	HNR_std	SD of HNR
	NHR_mean	Mean of NHR
	NHR_std	SD of NHR
**Nonlinear feature**		
	DFA	Detrended fluctuation analysis [20]
	RPDE	Recurrence period density entropy [21]
	D2	Correlation dimension [22]
	PPE	Pitch period entropy [23]

### Problem Formulation

#### Diagnosis

Because the predicted value in PD diagnosis is discrete and binary, it can be regarded as a two-category classification problem. This paper chose the following classical classification algorithms: (1) SVM, (2) ANN, (3) Naive Bayes, and (4) logistic regression.

#### Severity Assessment

Because the predicted value (ie, the UPDRS score) is continuous in the assessment of the severity of speech impairment in PD, it can be seen as a regression problem. This paper chose the following classical regression algorithms: (1) SVR, (2) linear regression, and (3) LASSO regression.

## Results

### Overview

We should initially introduce some indicators to evaluate the quality of the algorithms. First, for a two-category classification problem, there are usually the following classification results, as seen in Table 3.

**Table 3:** Classification confusion matrix.

Class	Predictive class	Predictive negative class
Actual positive class	True positive (TP)	False negative (FN)
Actual negative class	False positive (FP)	True negative (TN)

Then, the indicators are generally employed to evaluate the classification effect (see Figure 1, equations 4-7). The accuracy represents the proportion of subjects who are classified correctly out of the total number of subjects; precision indicates the proportion of real patients who are predicted to be sick; recall indicates the proportion of patients who are predicted to be sick; and the F1 value is the harmonic mean of the accuracy rate and the recall rate. In our PD diagnosis task, if a normal user is detected to be sick, the impact is usually not large, since we can continue to check the result using various clinical methods. However, if a model fails to detect PD, the impact is relatively large. Hence, the most important indicator is the recall rate.

Second, for a regression problem, if the total number of samples is *N*, the true value of the *i*-th sample is *T_i_*, and the predicted value is *P_i_*, then the indicators in equations 8-10 (see Figure 1) are available. Among the indicators, mean absolute error (MAE) measures the average magnitude of the errors in a set of predictions, without considering their direction; mean square error (MSE) and root mean square error (RMSE) are quadratic scoring rules that also measure the average magnitude of the error. However, both MSE and RMSE give a relatively higher weight to large errors. As a result, they are more useful when large errors are particularly undesirable.

According to the characteristics of the dataset, different experiments were performed on the three kinds of datasets downloaded from UCI. The characteristics of these datasets are shown in Table 4.

**Table 4:** Characteristics of three datasets from the University of California Irvine.

Data characteristics		Dataset 1	Dataset 2	Dataset 3
Creation date (year/month/day)		2008/06/26	2014/06/12	2009/10/29
**Number of subjects**				
	Parkinson disease	23	48	42
	Non-Parkinson disease	8	20	0
Number of records (ie, samples)		195	1208	5875
Number of features		22	26	18
Task		Classification	Classification and regression	Regression

All results are based on experiments with 5-fold cross validation. To evaluate our models’ efficiency and effectiveness, for the PD diagnosis (ie, classification task), the ratio of the training set to the validation set was 4:1 in the first two datasets. We then used a dataset collected from a local hospital as the test dataset. The data collection table is shown in Multimedia Appendix 1. We collected a dataset that included 14 PD patients and 30 non-PD patients in total. For the PD severity evaluation (ie, regression task), the ratio of training set to the validation set to the testing set was 4:1:1 in the third dataset. The testing results are shown in the following paragraphs.

For the first set of data [9], we conducted classification experiments according to a combination of linear and nonlinear features; the final result is shown in Table 5.

**Table 5:** Classification results for the first set of data.

Algorithm	Accuracy (%)	Precision (%)	Recall (%)	F1 score (%)
Support vector machine	*88.74* ^a^	88.89	*97.03*	92.55
Logistic regression	85.71	89.97	91.32	90.18
Neural network (single layer)	88.68	91.16	94.26	92.45
Neural network (double layer)	88.63	92.55	93.38	*92.71*
Naive Bayes	69.24	*96.02*	62.37	75.21

^a^Italics represent the highest values.

We can see that the combination of linear and nonlinear features for the diagnosis of PD patients is feasible and effective. The SVM algorithm achieved higher accuracy and recall rate, and the Naive Bayes algorithm had the worst effect. According to the previous discussion, the recall rate is the most important indicator. At the same time, considering the speed requirement of the mobile app, our system finally leveraged the SVM algorithm to perform the PD patient diagnosis. From Multimedia Appendix 2, we can see that these features have small *P* values, especially for the nonlinear features, which statistically show the effectiveness of these features.

For the second set of data [24], we conducted classification experiments using only linear features, and the final result is demonstrated in Table 6.

**Table 6:** Classification results for the second set of data.

Algorithm	Accuracy, %	Precision, %	Recall, %	F1 score, %
Support vector machine	66.71	66.37	*83.71* ^a^	73.98
Logistic regression	66.56	67.68	79.08	72.84
Neural network (single layer)	*70.78*	71.13	81.54	*75.89*
Neural network (double layer)	70.29	*71.45*	80.81	75.40
Naive Bayes	59.36	61.80	73.78	67.19

^a^Italics represent the highest values.

It can be clearly seen that using only linear features for PD diagnosis brings about a poor model performance, which is consistent with the conclusion from Tsanas et al [11] that feeding linear features into speech models is not very satisfactory. Meanwhile, some researchers claimed that nonlinear features are more effective [23], and another PD speech dataset analysis study [24] also obtained similar results. In particular, our experimental results showed that the SVM algorithm achieved a relatively high recall rate.

For the third set of data (ie, regression) [25], we tested multiple regression algorithms on the third dataset. The final result is illustrated in Table 7.

**Table 7:** Regression results on the third dataset.

Algorithm	Mean absolute error	Mean square error	Root mean square error
Linear regression	8.0786	95.1344	9.7494
Support vector machine	*3.7699* ^a^	*34.1202*	*5.8357*
Least absolute shrinkage and selection operator	8.0687	91.1600	9.7452

^a^Italics represent the best values.

Experimental results showed that both linear and nonlinear features contribute to the severity assessment of PD patients. Among regression algorithms, the SVR algorithm achieved the best performance on each indicator, and the prediction results of LASSO and linear regressions were not much different; the reason for this is that LASSO regression is actually a variant of linear regression. Hence, the system finally adopted SVR as the severity evaluation method.

**Figure 3:**
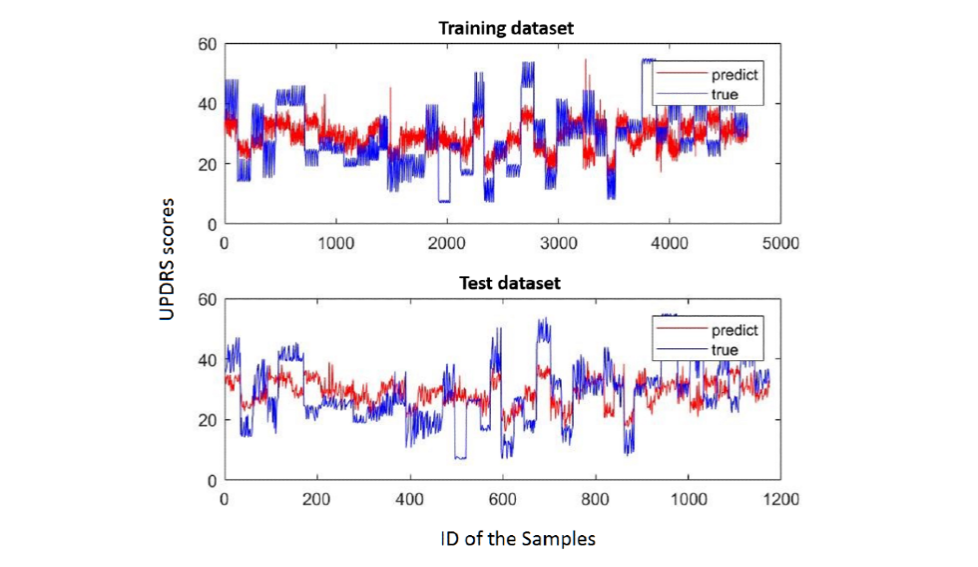
Linear regression fitting. The red line is the predicted value and the blue line is the true value. UPDRS: Unified Parkinson's Disease Rating Scale.

**Figure 4:**
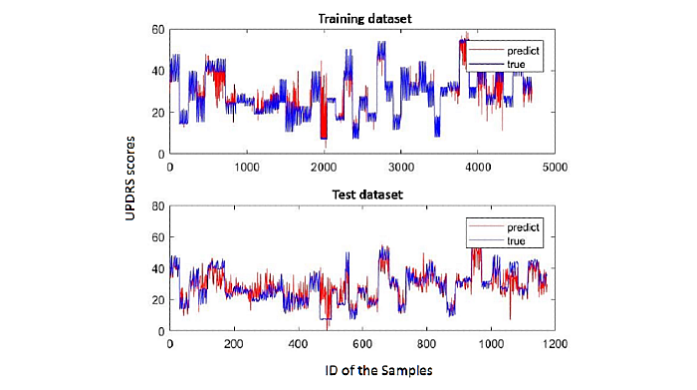
Support vector regression (SVR) fitting. The red line is the predicted value and the blue line is the true value. UPDRS: Unified Parkinson's Disease Rating Scale.

**Figure 5:**
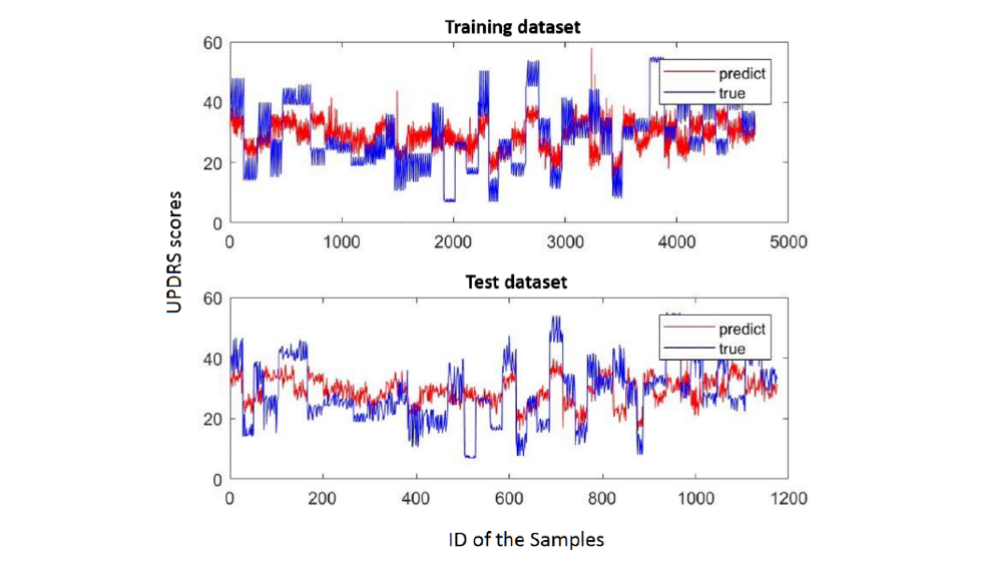
Least absolute shrinkage and selection operator (LASSO) fitting. The red line is the predicted value and the blue line is the true value. UPDRS: Unified Parkinson's Disease Rating Scale.

In particular, we selected the best results from each algorithm and observed the degree of fit. Figures 3-5 show the fitting results of the aforementioned three methods. In each figure, the upper graph is the degree of fitting of the training set and the lower graph is the degree of fitting of the test set; the red line is the predicted value and the blue line is the true value. It can be seen from these three figures that SVR fits the best.

As we know, LASSO can perform feature selection [26] by setting the feature weights to zero. The five characteristics most relevant to the value are shown in Table 8.

**Table 8:** Top five principal characteristics.

Feature	Corresponding weighted value
Age	2.84
Harmonics-to-noise ratio mean	–2.66
Absolute jitter	–2.18
Detrended fluctuation analysis	2.14
Pitch period entropy	1.51

It can be considered that these five characteristics are highly correlated with the UPDRS score. Age itself is highly related to PD, and the rest of the characteristics have three nonlinear features—HNR mean is also a nonlinear feature—indicating the importance of nonlinear features. Gender also explains why the regression result of the second set of data was relatively poor.

From Multimedia Appendix 2, we see that these features all have small *P* values—the features of the Jitter series may be a bit higher than others—which proves that we need these features for our system.

In summary, the PD speech detection system uses SVM and SVR for PD speech diagnosis and severity assessment, respectively.

### System

#### System Overview

**Figure 6:**
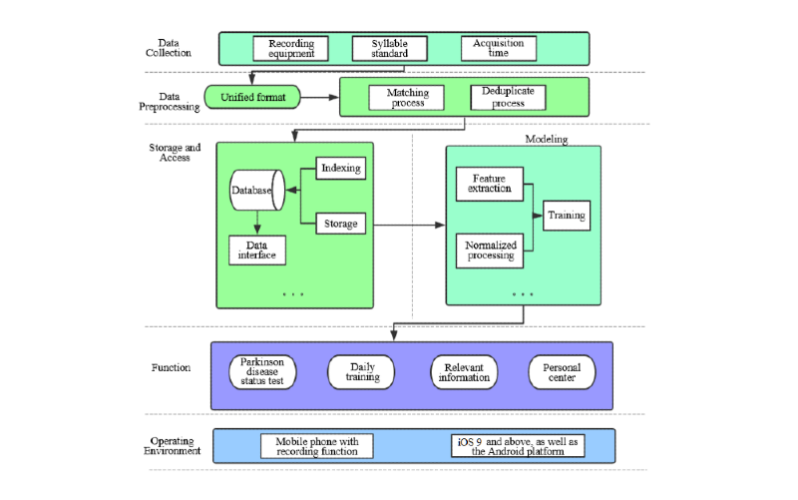
Architecture overview of the No Pa app system.

Figure 6 shows the architecture of our app system—called the No Pa app—including voice signal collection, data preprocessing, data storage and access, and signal modeling. At its core, the PD diagnosis model is SVM trained by the first set of data and the PD severity assessment model is SVR trained by the third set of data. Meanwhile, Figure 6 displays the four key functions and the operating environment in the application layer.

#### The Main Function

**Figure 7:**
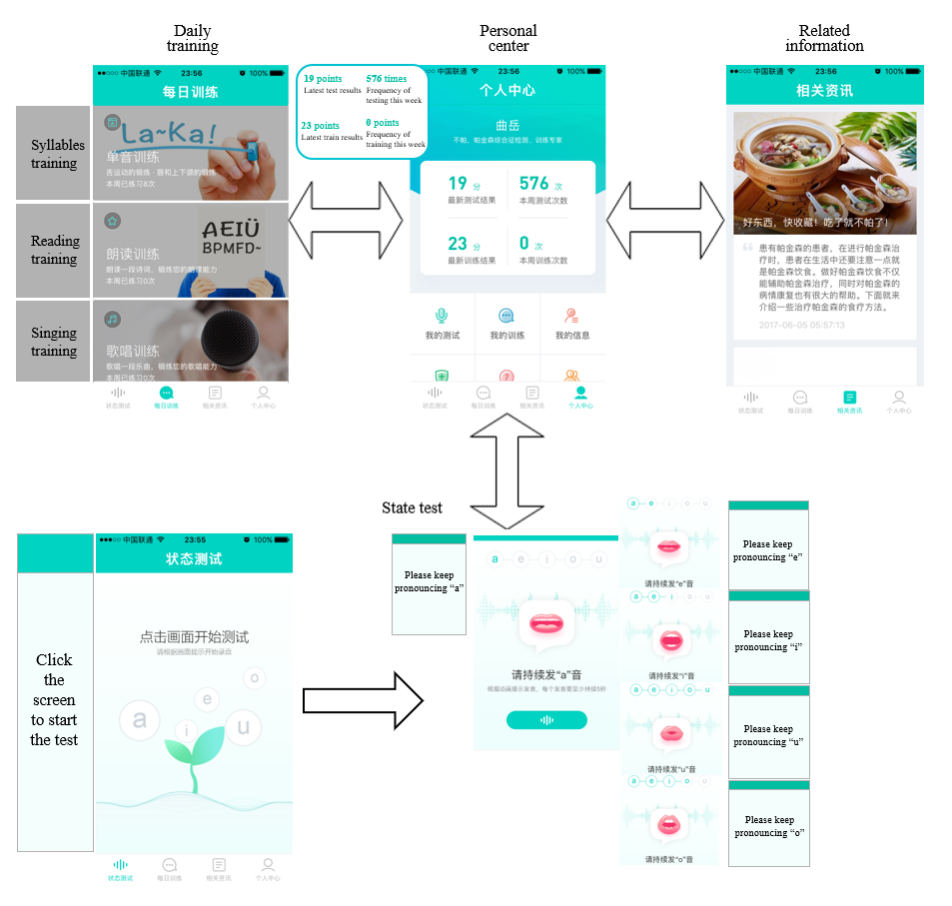
Screen captures from the No Pa app showing four functional modules.

Android and iOS versions of the No Pa mobile app are currently available online. The app includes four functions—state test, daily training, related information, and personal center—which are shown as follows (see Figure 7 for a few screen captures):

1. State test: the subject pronounces five long vowels according to the voice guidance, and each long vowel sound lasts for 5 seconds. Then, our system will calculate the current speech impairment severity status.
2. Daily training: the daily training function aims to improve subjects’ speech impairment status by encouraging them to speak. It includes monophonic training, reading training, and singing training. Monophonic training includes the user's pronunciation training according to some specific single syllables; during reading training, the user reads ancient poetry; and singing training improves the user's daily training interest via singing songs. Note that each training function will give a corresponding feedback score according to our speech signals model. However, since the calculation is not based on the five long vowels, the scores may not be accurate, but it is acceptable since our aim is to attract subjects’ attention to daily training in speaking.
3. Related information: this function provides users with some advice about PD and physical health.
4. Personal center: this function helps the user view their testing history and some personal information.

#### Back-End Configuration

The back-end server of the No Pa app is the Alibaba Cloud Server. Its configuration is as follows: 4-core central processing unit (CPU), 8 GB RAM, 64-bit Ubuntu system, and 200 GB disk space.

#### Algorithm Acceleration

The original system’s computational cost can range from 20 to 30 seconds without any acceleration techniques. Experimental results showed that autocorrelation calculation is the most time-consuming unit, so the C++ programming language was used to accelerate the autocorrelation calculation. To speed up the system, we adopted MEX (MATLAB executable) technology [27] as the acceleration scheme. In the end, the computational cost for predicting UPDRS scores was compressed from 20 seconds to only about 1 second. This response time is acceptable for an app.

#### Guide and Interaction

For better a user experience, we provided voice-guided navigation that can offer step-by-step instructions. Meanwhile, considering that PD patients may suffer from hand tremors, we designed big buttons in this app. Moreover, if they do not click the recording function button or the system fails to record an effective sound, the system will give them a reminder.

## Discussion

### Principal Findings

Traditionally, PD patients need to be diagnosed by physical examination. We can now use a mobile app to help conduct straightforward and rapid detection. For PD patients or healthy people, instant detection and consistent monitoring of disease conditions are extremely important. For doctors, the app can be used as a decision-support tool to provide assistance in treatment and diagnosis.

We have built this mobile app by embedding a voice-oriented system. At the core of the system are machine learning algorithms. Experimental results showed that SVM and SVR achieved the best performance for the diagnosis (ie, classification task) and severity evaluation (ie, regression task) of PD, respectively. The recall rate of the classification task can reach 97.03% (ie, the patient's recognition ability), and the absolute average error of the regression task can reach 3.7699, which is acceptable since the value of UPDRS scores range from 0 to 199.

Finally, we will summarize the contributions of our work. We have built a voice-oriented system that can remotely and conveniently diagnose PD. The system first collects a user’s five long vowels and then efficiently extracts dysphonia features, such that machine learning algorithms can be applied to the classification or regression of PD-related tasks. First, the system has been integrated into an app for public use. Second, our experiments have validated the effectiveness of voice signal–related features proposed by mainstream studies. Third, our system incorporates voice signal collection, feature extraction, and an algorithm interface, which can be regarded as a standard open-source platform for new algorithm development in voice signal–oriented disease identification tasks.

### Comparison With Prior Work

There have been various studies utilizing vocal features for PD diagnosis or severity evaluation. More specifically, Lahmiri et al [7] proposed a study about diagnosing PD based on dysphonia measures. They chose the same dataset as our first dataset and their results are similar to ours. However, our method achieved a higher recall value on this dataset. Wroge et al [8] also focused on PD diagnosis by speech signal analysis. After some speech signal processing, they extracted two groups of features—Audio-Visual Emotion recognition Challenge (AVEC) [12] and Geneva Minimalistic Acoustic Parameter Set (GeMAPS) features [13]—which were then fed into some machine learning models. However, their feature extraction process relied on some existing tools, which are not easily integrated into an app. In particular, their work needs to extract 1262 features while our work only extracts 24 features. Moreover, the accuracy of their results based on SVM and ANN were both lower than ours. Similar work that is based on the above features can be found in Tracy et al [28]. Deep learning methods have also been leveraged to learn patterns from vocal feature sets [29]. However, their model lacks explanations due to the inherent nature of deep learning models and achieves an inferior performance compared with our model. Moreover, besides PD diagnosis, our system realizes PD severity evaluation, which may be more helpful for patients and doctors.

### Limitations

Our data were collected from healthy people and patients with PD from Dalian, China; the quantity of data is still not big enough. In the future, we plan to collect more disease-related data from different regions worldwide to improve the generalization of the model. At the same time, we will use deep learning methods to study the speech signals of patients with PD to avoid cumbersome manual extraction of speech signals.

## Appendix

Multimedia Appendix 1 Data collection table.

Multimedia Appendix 2 *P* values for linear and nonlinear features.

## Abbreviations

ANN: artificial neural network
AVEC: Audio-Visual Emotion recognition Challenge
CERNET: China Education and Research Network
COVID-19: coronavirus disease 2019
CPU: central processing unit
GeMAPS: Geneva Minimalistic Acoustic Parameter Set
HNR: harmonics-to-noise ratio
LASSO: least absolute shrinkage and selection operator
MAE: mean absolute error
MEX: MATLAB executable
MSE: mean square error
PD: Parkinson disease
RMSE: root mean square error
SVM: support vector machine
SVR: support vector regression
UCI: University of California Irvine
UPDRS: Unified Parkinson Disease Rating Scale
